# Accuracy of self-collected vaginal dry swabs using the Xpert human papillomavirus assay

**DOI:** 10.1371/journal.pone.0181905

**Published:** 2017-07-27

**Authors:** Rosa Catarino, Pierre Vassilakos, Aline Bilancioni, Stéphanie Bougel, Meriem Boukrid, Ulrike Meyer-Hamme, Patrick Petignat

**Affiliations:** 1 Division of Gynaecology, Department of Gynaecology and Obstetrics, Geneva University Hospitals, Geneva, Switzerland; 2 Geneva Foundation for Medical Education and Research, Geneva, Switzerland; 3 Biopath Lab SA, Lausanne, Switzerland; Victorian Cytology Service Ltd, AUSTRALIA

## Abstract

**Background:**

Polymerase chain reaction-based Xpert human papillomavirus (HPV) assay is a rapid test that detects high-risk HPV (hrHPV) infection. This point-of-care test is usually performed by collecting a cervical specimen in a vial of PreservCyt® transport medium. We compared HPV test positivity and accuracy between self-collected sample with a dry swab (s-DRY) versus physician-collected cervical sampling using a broom like brush and immediate immersion in PreservCyt (dr-WET).

**Methods:**

In this cross-sectional study, we recruited 150 women ≥ 18 years old attending the colposcopy clinic in the University Hospital of Geneva. Each participant first self-collected a vaginal sample using a dry swab and then the physician collected a cervical specimen in PreservCyt. HPV analysis was performed with Xpert. Part of the PreservCyt-collected sample was used for hrHPV detection with the cobas® HPV test. HPV test positivity and performance of the two collection methods was compared.

**Results:**

HPV positivity was 49.1% for s-DRY, 41.8% for dr-WET and 46.2% for cobas. Good agreement was found between s-DRY and dr-WET samples (kappa±Standard error (SE) = 0.64±0.09,), particularly for low-grade squamous intraepithelial lesions (LSIL+) (kappa±SE = 0.80±0.17). Excellent agreement was found between the two samples for HPV16 detection in general (kappa±SE = 0.91±0.09) and among LSIL+ lesions (kappa±SE = 1.00±0.17). Sensitivities and specificities were, respectively, 84.2% and 47.1%(s-DRY), 73.1% and 58.7%. (dr-WET) and 77.8% and 45.7% (cobas) for CIN2+ detection. The median delay between sampling and HPV analysis was 7 days for the Xpert HPV assay and 19 days for cobas. There were 36 (24.0%) invalid results among s-DRY samples and 4 (2.7%) among dr-WET (p = 0.001). Invalid results happened due to the long interval between collection and analysis.

**Conclusion:**

Self-collected vaginal dry swabs are a valid alternative to collecting cervical samples in PreservCyt solution for HPV testing with the Xpert HPV assay.

**Impact:**

HPV self-collection with dry cotton swabs might assist in the implementation of an effective screening strategy in developing countries.

**Trial registration:**

International Standard Randomized Controlled Trial Number Registry ISRCTN83050913

## Introduction

In recent years, the development of high-risk (hr) human papillomavirus (HPV) tests has created an important change in our approach to cervical cancer (CC) screening. Overwhelming evidence from several randomized trials has shown that HPV screening is more effective than cytology in preventing CC, with better sensitivity and less frequent screening [1, 2].

Developed countries are progressively incorporating HPV testing into their national screening programs and updating their current guidelines [3]. Developing countries, following the recommendations of the World Health Organization, are evaluating HPV testing as a primary screening tool [4]. HPV testing is associated with decreased CC-related mortality [5, 6] and it offers the possibility to perform self-collection of vaginal samples (self-HPV).

The introduction of a rapid point-of-care non-batch assay, which facilitates same-day screen and management strategies, is essential in developing countries, especially for screen-and-treat strategies. This approach minimizes the need for repeated visits, encouraging greater numbers of eligible women to participate in the program. The ideal system should be modular and easily integrated into low and medium-income countries (LMIC). Furthermore, real-time polymerase chain reaction (PCR)-based hrHPV tests with a high analytic sensitivity are preferable to ensure similar accuracy between clinician- and self-collected samples [7].

The Xpert HPV assay from Cepheid (Sunnyvale, CA, USA) is a non-batch qualitative real-time PCR assay that has the capability to perform point of care hrHPV testing. The assay is formatted in a single-use test cartridge and can be completed in 1 hour, meaning it can be used in a screen-and-treat strategy adapted to the LMIC context.

Xpert was previously evaluated for CC screening with specimens collected into a vial of PreservCyt® transport medium (Thinprep, Hologic, Bedford, MA, USA) [8–10]. However, besides the logistic issues of introducing cytology in LMIC, liquid-based cytology media are expensive and unavailable in a resource-poor context. Additionally, they are toxic and flammable, and spillage and leakage can occur during collection and transit.

In a previous self-HPV study we found that swabs transported in a dry state provided test results that were comparable to those obtained with swabs shipped in a wet transport medium, in terms of quality of results [11]. The possibility of using self-collection specimens stored at ambient temperature without transport media would clearly enhance and simplify self-HPV.

The aim of this study was 1) to compare HPV test positivity between self-HPV with a dry swab (s-DRY) versus physician-collected cervical sampling using a broom like brush and immediate immersion in PreservCyt (dr-WET) and 2) to assess the accuracy and agreement between the two collection methods.

## Methods

### Study population

From March 4, 2015 to October 31, 2015, 150 women were consecutively recruited from the colposcopy clinic of Geneva University Hospitals, Switzerland. Patients who attend this clinic are usually women with abnormal cytological results, persistent HPV infection, or follow-up of an untreated CIN1 lesion. Women were eligible if 1) they were at least 18 years old, 2) they understood the study procedures, and 3) voluntarily agreed to participate by signing a written informed consent form. Pregnant women and those with a history of total hysterectomy were excluded. The Cantonal Human Research Ethics Commission of Geneva approved the study (October 30, 2014; CCER: 14–228).

The protocol for this trial and supporting STARD and TREND Statement checklists are available as supporting information; see S1 and S2 Checklists and S1 Protocol.

### Study design and procedure

In this cross-sectional study, all women completed a self-administered questionnaire on demographics data. Eligible women were asked to perform self-HPV (s-DRY). Each participant received a package containing a specimen collection cotton swab in a plastic tube and instructions for use. They were instructed to wash their hands before the procedure. The instructions stated to hold the plastic head of the swab and insert the cotton tip of the swab into the vagina, avoiding contact with the external genitalia, until it met resistance. Holding the swab inside the vagina it should be gently turned 3 to 5 full rotations. After taking out the swab from the vagina and always holding the swab from the plastic head it should be placed inside the plastic tube. Following the self-collection and before colposcopic examination, the physician collected a cervical specimen using a Cervex brush (Rovers Medical Devices B.V., Oss, Netherlands), which was immediately placed in PreservCyt (dr-WET).

The two specimens (s-DRY and dr-WET) were tested for the same pathogens (hrHPV) using the same diagnostic test (Xpert). In addition, a part of the PreservCyt-collected sample (dr-WET) was used for hrHPV detection with the Food and Drug Administration (FDA) approved cobas® HPV Test (*Roche* Diagnostics, Basel, *Switzerland*). Then, with the remaining PreservCyt sample, a cytological preparation was performed if a Pap smear result was not available from the last 3 months.

All patients were invited for colposcopic examination and a biopsy with endocervical brushing was performed if necessary.

This trial was registered at ISRCTN Registry as ISRCTN83050913 (for logistic reasons the trial was registered after the recruitment began; the authors confirm that all ongoing and related trials for this intervention are registered). The study protocol is available in S1 Protocol.

### Laboratory methods

#### HPV detection with the Xpert HPV assay

After specimen collection was performed in the colposcopy clinic, both samples were stored at room temperature before being analyzed. In some cases, sampling and analysis was not performed on the same day, since the Xpert instrument only had 3 operative modules.

In the laboratory, dry swabs (s-DRY) were rinsed into tubes with 3 ml of 0.9% NaCl and vortexed 3 times for 15 seconds each. Then, 1 ml of each sample was transferred to the cartridge and a four-module Xpert assay was performed. PreservCyt vials containing dr-WET samples were also vortexed 3 times for 15 seconds each. Next, 1 ml of each sample was transferred to the cartridge and an Xpert assay was performed. The s-Dry and dr-WET aliquots from each patient were simultaneously analyzed by the Xpert HPV Assay.

Xpert is a multiplexed real-time PCR test that targets the E6 and E7 oncogenes of 14 cancer-related HPV types. It uses as an internal assay control for specimen adequacy the detection of a human reference gene (*HMBS* [hydroxymethylbilane synthase]) and an internal Probe Check Control (PCC). The PCC verifies reagent rehydration, PCR tube filling in the cartridge, probe integrity and dye stability. Threshold (Ct) values reflect the relative number of cells in the sample. A lower Ct-value represents a relatively higher number of cells.

Xpert includes reagents for the simultaneous detection of 14 hrHPV types (HPV 16, 18, 31, 33, 35, 39, 45, 51, 52, 56, 58, 59, 66 and 68). The assay utilizes six fluorescent channels for the detection of individual types of HPV, groups of HPV, and the human reference gene. Each fluorescent channel has its own cut-off parameters for target detection/validity. These fluorescent channels detect: 1) HPV 16; 2) HPV 18 and HPV 45; 3) HPV 31, 33, 35, 52, and 58; 4) HPV 51 and HPV 59; 5) HPV 39, 56, 66, and 68; and 6) *HMBS*. If sufficient signal is detected by the human reference gene, the assay results are reported as an overall “positive” if any type of targeted HPV is detected. Additionally, HPV 16 and pooled HPV 18/45 and, collectively, the other high-risk HPV types detected by the assay are reported specifically as “positive” or “negative.”

#### HPV detection with the cobas HPV test

part of the sample immersed in PreservCyt (dr-WET) was tested for HPV DNA using cobas at Biopath Lab, Lausanne. DNA was extracted from 400 μL of each sample. The cobas is a qualitative real time PCR assay using the cobas R4800 System. The detection is based on amplification of the L1 gene using TaqMan probes. The human reference gene ß-globin is also detected. Samples were analyzed according to the algorithm of Roche Diagnostics. This test allows the specific identification of HPV 16 and HPV 18 and detects 12 pooled hrHPV genotypes (31, 33, 35, 39, 45, 51, 52, 56, 58, 59, 66 and 68) at clinically relevant infection levels.

#### Liquid-based cytology

Cytologic preparations were made using the ThinPrep (Hologic) technology for all women. Cytotechnologists read the slides and a pathologist verified abnormal results for a final diagnosis. The results were classified according to the Bethesda System for cervical cytology.

#### Histopathology and management

Histopathological preparations of biopsies and endocervical brushings were interpreted at the Department of Pathology and Immunology, University of Geneva. Patient management was provided according to the Swiss standards in the colposcopy clinic of Geneva.

### Statistical analyses

Data were analyzed using Stata Statistical Software Release 13 (StataCorp LP, College Station, TX, USA). Total agreement, percent positive agreement between s-DRY and dr-WET samples for all hrHPV types and each HPV channel, overall and stratified on pathological cytology, were measured. The Cohen's kappa coefficients were also determined. The proportion of positive agreement between paired s-DRY and dr-WET samples was calculated using 2a/(f1 + g1), where a is the number of samples that were positive for HPV in both tests, f1 is the number of samples that were positive for s-DRY and g1 is the number of samples that were positive for dr-WET.

We used low-grade squamous intraepithelial lesions or worse (LSIL+) as a cut-off, which includes LSIL, high-grade squamous intraepithelial lesions (HSIL), adenocarcinoma in situ, and invasive cervical cancer (CC). Sensitivity and specificity and positive and negative predictive values were also calculated for each method, using cobas results as the reference standard for hrHPV, since it is a FDA approved HPV test. Histological results (cervical intraepithelial neoplasia grade 2 or more severe, CIN2+) were used as the reference standard for cervical lesions. McNemar’s two-tailed test was used for mutual comparison of sensitivity and specificity. P values less than 0.05 were considered statistically significant. The mean Ct-values for the *HMBS* adequacy test were calculated for the two collection modalities. We also analyzed invalid test results from Xpert and the effect of a delay between self-collection and HPV analysis.

Kaplan-Meier curves were done to examine the association between the number of invalid HPV test results and the elapsed time for both s-DRY and dr-WET samples.

Invalid HPV tests were excluded in the agreement and accuracy analysis.

A sample size of 150 women was calculated to provide a 10% precision to estimate the kappa coefficient, if the kappa is 50% (worst case scenario, as the precision will be better if the kappa is lower or higher than 50%). Assuming a 40% prevalence of the HPV infection in our selected population, the precision of other measures will be more or less 15%.

## Results

### Sample characteristics

**Fig 1:**
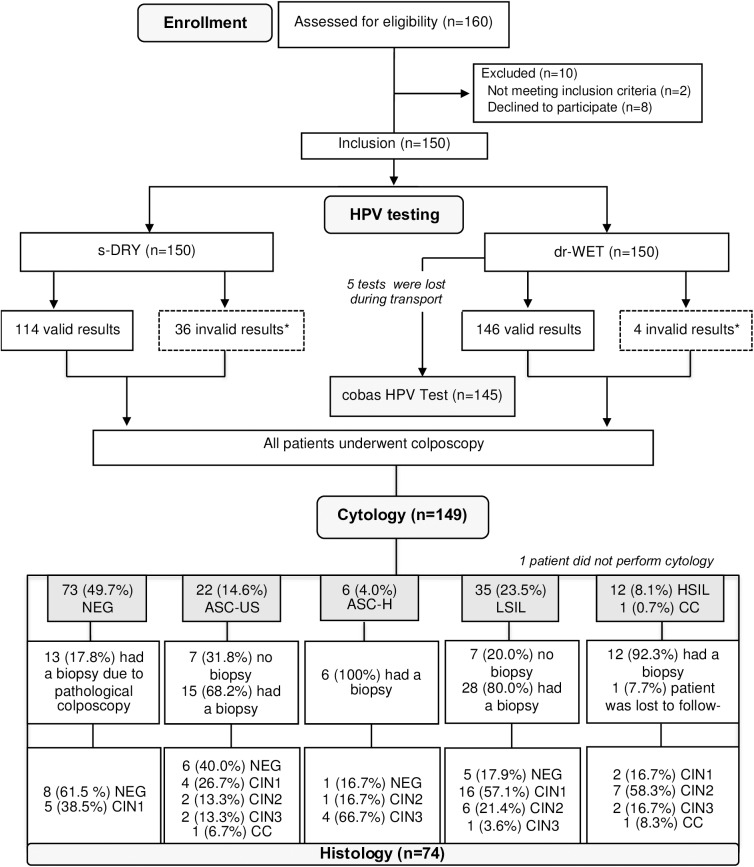
Flowchart of study participants. Abbreviations: dr-WET = physician-collected sample with swab immediately immersed in PreservCyt; s-DRY = Self-vaginal collection with dry swab. ASC-US = Atypical squamous cells of undetermined significance; ASC-H = Atypical squamous cells-cannot rule out high grade; LSIL = Low-grade squamous intraepithelial lesion; HSIL = High-grade squamous intraepithelial lesion; CIN1 = cervical intraepithelial neoplasia grade 1; CIN2 = cervical intraepithelial neoplasia grade 2; CIN3 = cervical intraepithelial neoplasia grade 3; CIN2+ = cervical intraepithelial neoplasia grade 2 or more severe. * Invalid results were excluded in the agreement analysis between dr-WET and s-DRY samples, as well as in the determination of clinical performance, using cobas results and histology (CIN2+), as reference standard.

Overall, 150 patients were included in the study. The study flowchart is shown in **Fig 1**. The median age of the participants was 32 years (interquartile range [IQR] 27–41), and the majority had a partner (80.0%). Sociodemographic data are represented in **Table 1**. The median delay between sampling and Xpert HPV assay analysis was 7 (IQR 2–23) days. The median delay between sampling and cobas HPV analysis was 19 (IQR 11–25) days. The greater delay between collection and analysis for the cobas test was due to the fact that the sample needed to be transported to another laboratory outside the Geneva University Hospitals in order to be processed. Moreover, the cobas is a batch test, which means that we needed to have enough samples to run the analysis. The Xpert HPV assay is a non-batch test and it was located next to the Colposcopy clinic, which facilitated the whole process.

**Table 1:** Sample sociodemographic characteristics and self-HPV assessment.

Variable	n (%)
Age, median (IQR), y	32 (27–41)
Age groups, y	
18–29	57 (38.0)
30–39	50 (33.3)
40–70	43 (28.7)
Marital Status	
Without a partner	30 (20.0)
With a partner	120 (80.0)
Children	
No	71 (47.3)
Yes	79 (52.7)
Median (IQR)	1 (0–2)
Education	
None	4 (2.7)
Primary education	35 (23.6)
Secondary education	51 (34.5)
Tertiary education	58 (39.2)
Self-HPV pain assessment	
Not painful at all	98 (65.3)
Slightly painful	37 (24.7)
Moderately painful	11 (7.3)
Very painful	4 (2.7)

Abbreviations: IQR: interquartile range n: number; y: years.

### Overall HPV detection according to method

HPV positivity was 56 (49.1%) for s-DRY samples, 61 (41.8%) for dr-WET samples and 67 (46.2%) for cobas. Among dr-WET samples, HPV positivity was 23 (15.8%) for HPV 16, 10 (6.9%) for HPV 18/45 and 28 (26.0%) for other hrHPV types. Among s-DRY samples, HPV positivity was 21 (18.4%) for HPV 16, 7 (6.1%) for HPV 18/45 and 34 (29.8) for other hrHPV types. Using the cobas test, HPV positivity was 25 (17.2%) for HPV 16, 5 (3.5%) for HPV 18 and 47 (32.4) for other hrHPV types.

The mean Ct-values for the β-globin gene were 25.1 ± 14.3 for s-DRY samples and 30.5 ± 5.0 for dr-WET samples (p < 0.001). A lower Ct-value represents a relatively higher number of cells.

**Fig 2:**
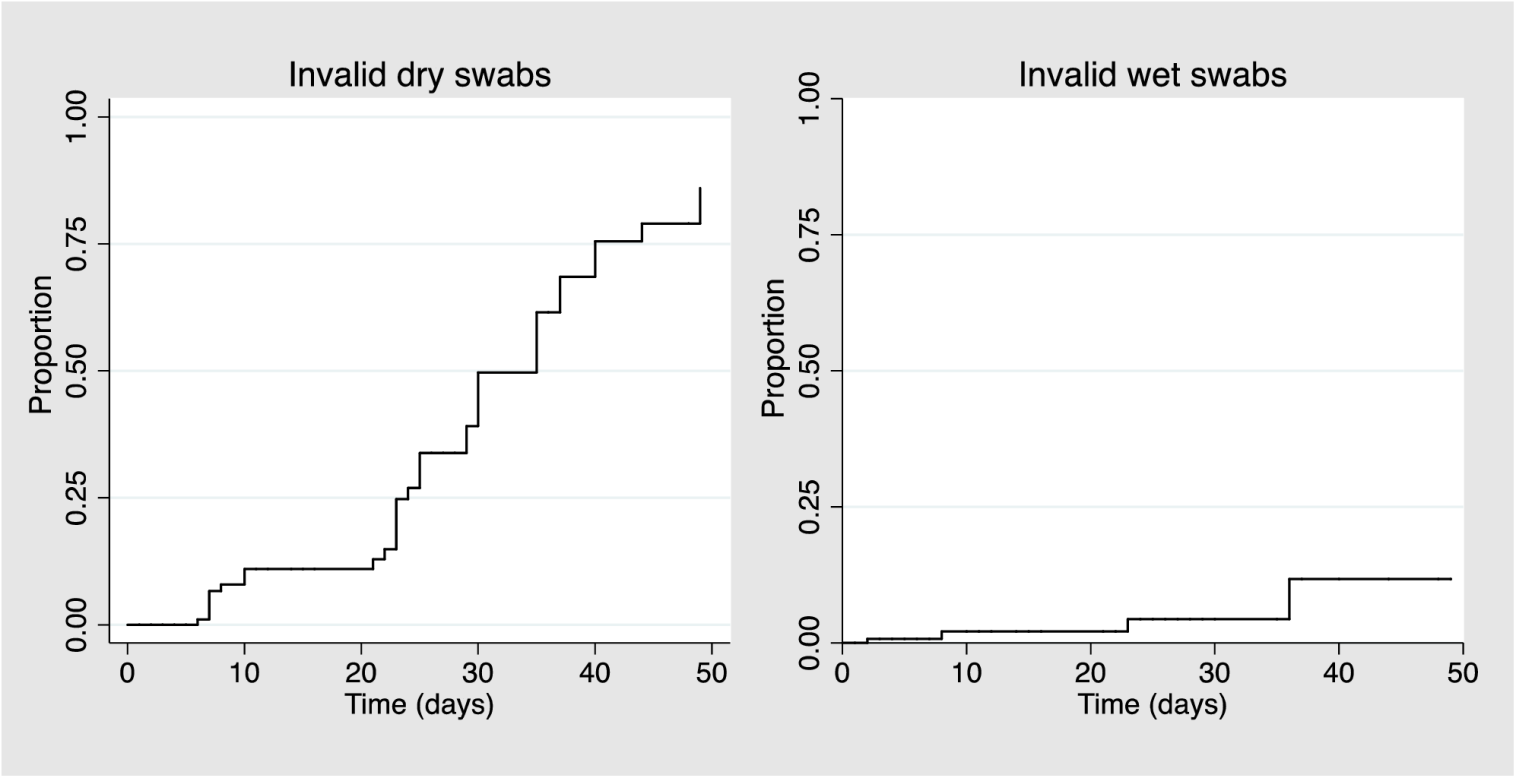
Kaplan–Meier failure estimate showing the proportion of s-DRY and dr-WET invalid tests by length of delay.

There were 36 (24.0%) invalid results among s-DRY samples and 4 (2.7%) among dr-WET (p = 0.001). The invalid results among s-DRY samples were due to the long interval between collection and analysis; all invalid results occurred in samples that waited 6 or more days before processing. A delay increased invalid results from 1.6% in the first week to 19.5% during the second week and 56.3% after more than 2 weeks (p < 0.001) among s-DRY samples. The median interval between sampling and analysis was 5.5 (2–10) days among valid results and 25 (15.5–35) days among invalid results (p < 0.001). In **Fig 2**, a Kaplan–Meier failure estimate shows a clear increase in the proportion of invalid tests with increasing time elapsed between s-DRY samples.

### Cytological and histological results

Among the 149 (99.3%) patients with cytological results, there were 73 (49.0%) with negative cytology, 22 (14.8%) with atypical squamous cells of undetermined significance (ASC-US), 6 (4.0%) with atypical squamous cells-cannot rule out high grade (ASC-H), 35 (23.5%) with LSIL, 12 (8.1%) with HSIL and 1 (0.7%) with CC (**Fig 1**). Forty-eight (32.2%) patients had LSIL+. A total of 74 (49.3%) patients were biopsied. There were 20 (27.0%) negative results, 27 (36.5%) CIN1, 16 (21.6%) CIN2, 9 (12.2%) CIN3 and 2 (2.7%) CC cases. Twenty-seven (36.5%) patients had CIN2+ (**Table 2**). The test positivity increased with increasing severity of diagnosis for both s-DRY and dr-WET methods.

**Table 2:** Distribution of histological diagnoses and the percentages of positive test results for hrHPV by s-DRY, dr-WET and cobas (n = 150, 74 biopsies, 76 without biopsy).

Diagnosis	No. Of tests	% of tests	s-DRY n (%) pos	dr-WET n (%) pos	cobas n (%) pos
Neg	20	27.0	6/15 (40.0)	7/20 (35.0)	9/20 (45.0)
CIN1	27	36.5	12/19 (63.2)	12/26 (46.2)	16/26 (61.5)
CIN2	16	21.6	7/9 (77.8)	9/16 (56.3)	11/16 (68.8)
CIN3	9	12.2	7/8 (87.5)	8/8 (100.0)	9/9 (100.0)
CC	2	2.7	2/2 (100.0)	2/2 (100.0)	1/2 (50.0)
CIN2+	27	36.5	16/19 (84.2)	19/26 (73.1)	21/27 (77.8)
Total number of biopsies	74	100.0			
Negative colposcopy (No biopsy)	76	50.7	36.1	31.1	29.2

Abbreviations: n: number; CC: Cervical Cancer.

### Agreement between collection methods according to cytological results

**Table 3** represents the agreement, overall and stratified by cytological results (LSIL+ vs < LSIL), for hrHPV detection and for each HPV channel. The kappa (95% CI) between s-DRY and dr-WET for hrHPV was 0.64 (0.50–0.78) in general, 0.53 (0.34–0.73) among those with less than LSIL and 0.80 (0.59–1.00) among LSIL+ cases. The kappa was higher for the detection of HPV 16: 0.91 (0.80–1.00) in general, 0.84 (0.65–1.00) among those with less than LSIL and 1.00 among LSIL+ cases. The kappa was also excellent among LSIL+ cases for the detection of HPV 18/45 (1.00).

**Table 3:** Agreement between s-DRY and dr-WET for Xpert HPV results for all hrHPV and each HPV channel, overall and stratified on disease status, negative cytology, and LSIL or more severe diagnoses (LSIL+).

HPV Category	s-DRY+/ dr-WET+		s-DRY+/ dr-WET-		s-DRY-/ dr-WET+		s-DRY-/ dr-WET-		Total		κ (95% CI)	% Agreement	% Positive Agreement*
	n	%	n	%	n	%	n	%	n	%			
All													
hrHPV	42	37.8	12	10.8	8	7.2	49	44.1	111	100	0.64 (0.50–0.78)	82	80.8
HPV16	18	16.2	2	1.8	1	0.9	90	81.1	111	100	0.91 (0.80–1.00)	97.3	92.3
HPV18, 45	5	4.5	2	1.8	2	1.8	102	91.9	111	100	0.70 (0.41–0.98)	96.4	71.4
HPV31, 33, 35, 52, 58	12	10.8	6	5.4	6	5.4	87	78.4	111	100	0.60 (0.40–0.81)	89.2	66.7
HPV51, 59	5	4.5	3	2.7	3	2.7	99	89.2	111	100	0.60 (0.30–0.89)	94.6	62.5
HPV39, 56, 66, 68	4	3.6	4	3.6	4	3.6	99	89.2	111	100	0.46 (0.14–0.78)	92.8	50
<LSIL													
hrHPV	21	27.3	10	13	7	9.1	39	50.6	77	100	0.53 (0.34–0.73)	77.9	71.2
HPV16	9	11.7	2	2.6	1	1.3	65	84.4	77	100	0.84 (0.65–1.00)	96.1	85.7
HPV18, 45	1	1.3	2	2.6	2	2.6	72	93.5	77	100	0.31 (0.19–0.81)	94.1	33.3
HPV31, 33, 35, 52, 58	7	9.1	6	7.8	4	5.2	60	77.9	77	100	0.51 (0.24–0.77)	87	58.3
HPV51, 59	2	2.6	2	2.6	2	2.6	71	92.2	77	100	0.47 (0.03–0.91)	94.8	50
HPV39, 56, 66, 68	1	1.3	2	2.6	3	3.9	71	92.2	77	100	0.25 (0.20–0.70)	93.5	40
LSIL+													
hrHPV	20	60.6	2	6.1	1	3	10	30.3	33	100	0.80 (0.59–1.00)	90.9	93
HPV16	9	27.3	0	0	0	0	24	72.7	33	100	1.00 (1.00–1.00)	100	100
HPV18, 45	4	12.1	0	0	0	0	29	87.9	33	100	1.00 (1.00–1.00)	100	100
HPV31, 33, 35, 52, 58	5	15.2	0	0	2	6.1	26	78.8	33	100	0.80 (0.53–1.00)	93.9	83.3
HPV51, 59	3	9.1	1	3	1	3	28	84.8	33	100	0.72 (0.34–1.00)	93.9	75
HPV39, 56, 66, 68	2	6.1	2	6.1	1	3	28	84.8	33	100	0.52 (0.05–0.99)	90.9	57.1

Abbreviations: s-DRY: HPV self-collection using a dry swab; dr-WET: physician-collected sample using PreservCyt; LSIL+: Low-grade squamous intraepithelial lesion or worse; n = number.

*The proportion of positive agreement between paired s-DRY and dr-WET samples was calculated using 2a/(f1 + g1), where a is the number of samples that were positive for HPV in both tests, f1 is the number of samples positive for s-DRY and g1 is the number of samples positive for dr-WET.

Overall, the agreement ranged from 82.0% to 97.3% and the positive agreement ranged from 50.0% to 92.3%. Among those with less than LSIL, the agreement ranged from 77.9% to 96.1% and the positive agreement ranged from 33.3% to 85.7%. Among those with LSIL+, the agreement ranged from 90.9% to 100% and the positive agreement ranged from 57.1% to 100%.

Overall, 111 women had a valid s-DRY and dr-WET test result. Three women had s-DRY valid test but dr-WET invalid, 35 women had dr-WET valid test but s-DRY invalid and 1 woman had both tests invalid.

### Clinical performance of self-collection and physician-collection methods

Sensitivity of the s-DRY method for hrHPV using the cobas results as the reference standard was 79.3% (95% CI: 65.8–88.3), compared with 78.8% (95% CI: 50.7–67.0) for the dr-WET method (p = 0.763). We found specificities of 80.4% (95% CI: 67.4–89.0) and 93.3% (95% CI: 84.7–97.3) (p = 0.034) for the s-lDRY and dr-WET methods, respectively (**Table 4**).

**Table 4:** Clinical performance of self-collection and physician-collection methods.

Variables	s-DRY and dr-WET performances, using cobas as gold-standard			
	Sensitivity (95% CI)	Specificity (95% CI)	PPV (95% CI)	NPV (95% CI)
s-DRY	42/53, 79.3% (65.8–88.3)	45/56, 80.4% (67.4–89.0)	42/53, 79.3% (65.8–88.3)	45/56, 80.4% (67.4–89.0)
dr-WET	52/66, 78.8% (65.8–86.2)	70/75, 93.3% (84.7–97.3)	52/57, 91.2% (80.1–96.4)	70/84, 83.3% (73.6–90.0)
p value*	0.763	0.034	.	.
	s-DRY and dr-WET performances, using histology as gold-standard (CIN2+)			
	Sensitivity (95% CI)	Specificity (95% CI)	PPV (95% CI)	NPV (95% CI)
s-DRY	16/19, 84.2% (57.8–95.4)	16/34, 47.1% (30.4–64.4)	16/34, 47.1% (30.4–64.4)	16/19, 84.2% (57.8–95.4)
dr-WET	19/26, 73.1% (51.7–87.3)	27/46, 58.7% (43.6–72.3)	19/38, 50.0% (33.9–66.1)	27/34, 79.4% (61.6–90.3)
p value*	0.563	0.317		
Cobas	21/27, 77.8% (57.0–90.2)	21/46, 45.7% (31.5–60.5)	21/46, 45.7% (31.5–60.5)	21/27, 77.8% (57.0–90.2)
p value (vs. s-DRY)*	0.998	0.527		
p value (vs. Dr-WET)*	0.564	0.035	.	.

Abbreviations: CI: Confidence interval

*The p-value was calculated with McNemar's test

Using the histological results as the reference standard for CIN2+, the s-DRY had a sensitivity of 84.2% (95% CI: 57.8–95.4) and specificity of 47.1% (95% CI: 30.4–64.4); dr-WET sensitivity was 73.1% (95% CI: 51.7–87.3) with specificity of 58.7% (95% CI: 43.6–72.3).

## Discussion

The Xpert HPV assay has been previously evaluated [8–10]. To our knowledge, this is the first study to assess the performance of self-collection with dry cotton swabs for HPV testing with the Xpert HPV assay. Our results suggest that dry swabs performance is similar to the performance of clinically validated PreservCyt-collected samples.

HPV positivity was higher for vaginal dry samples than cervical wet samples (49.1% vs 41.8%). The mean Ct-values for the β-globin gene were 25.1 ± 14.3 for s-DRY samples and 30.5 ± 5.0 for dr-WET samples, which means that dry samples had a relatively higher number of cells comparing to wet samples (p < 0.001). Moreover, good agreement was found between s-DRY and dr-WET samples. The agreement improved in pathological cytology cases (LSIL+). Better agreement between the two samples was also found for HPV 16 and HPV 18/45. Agreement was only fair between the two samples for genotypes 39, 56, 66 and 68. These results are consistent with previous studies comparing dry and wet samples using different HPV DNA tests such as the Roche Linear Array [12], Seegene Anyplex II HPV28 [13] and Roche Cobas 4800 test [14].

Dry samples had a clinical performance similar in terms of sensitivity to wet samples for hrHPV using cobas as the reference standard. However, for detecting CIN2+, there was a trend towards a better performance with s-DRY as compared to dr-WET or cobas, although these differences did not reach statistical significance. Equivalent and even improved sensitivity of self-HPV was found in previous studies when PCR-based HPV tests were used [13, 15, 16]. Our results are consistent with these studies, with a specificity of s-DRY that is lower than dr-WET samples.

We acknowledge some limitations of our study. We enrolled a population of women referred to the colposcopy clinic due to abnormal cytological results, persistent HPV infection, or follow-up of an untreated CIN1 lesion. This led to a high HPV prevalence [17], resulting in a higher rate of agreement between the two samples than would be expected in the general screening population [8, 18, 19]. Another limitation was that the dry samples were collected before the physician-collected wet samples, which may potentially provide an advantage to the first test. Finally, the Xpert HPV assay was not always used as a point of care test, since there was a significant delay between sample collection and HPV analysis, which resulted in number of invalid results among s-DRY samples. This is consistent with a previous study performed in field conditions in Madagascar, where dry swabs stored at room temperature were used [20].

In the present study, invalid results increased as the storage interval increased, from 1.6% in the first week to 19.5% during the second week and 56.3% after more than 2 weeks (p < 0.001). Consequently, dry samples should be analysed within 1 week of collection in order to guarantee valid results. Wet samples were much less affected by a delay, with only four invalid samples, which is not surprising since PreservCyt medium is known for its long-term stability [21]. The Xpert assay is a point of care HPV test that is intended for use in a "screen and treat” strategy in developing countries and therefore a delay between collection and analysis is unlikely to occur in actual use.

The strengths of our study include the ability to compare the s-DRY and dr-WET samples within categories of cytological and histological diagnoses. Moreover, we have also assessed agreement according to HPV channel and evaluated the performance of s-DRY, dr-WET and cobas among women with CIN2+ diagnoses.

In conclusion, our data indicate that HPV self-collection with dry cotton swabs is a valid alternative to wet-collected cervical samples for the Xpert HPV assay if the samples are tested within a short period (less than 1 week) and might assist in the implementation of an effective screening strategy in developing countries. Further validation with real-world testing and large population-based prospective cohort studies is warranted to confirm our results.

## Supporting information

S1 Checklist TREND statement checklist. (PDF)

S2 Checklist STARD checklist. (DOCX)

S1 Protocol Study protocol. (PDF)
