# Supplementary material for: Accuracy of self-collected vaginal dry swabs using the Xpert human papillomavirus assay
Source: PLoS One. 2017 Jul 27;12(7):e0181905. doi: 10.1371/journal.pone.0181905 (PMC5547698; doi:10.1371/journal.pone.0181905)
Supplement: S1 Protocol — (PDF) [file pone.0181905.s003.pdf]

Validation Study of vaginal dry swabs using the Xpert HPV test for human papillomavirus diagnosis

Study Protocol

September 2015

Version 3

Main investigator:

Rosa Catarino, Gynecology service, HUG

RosaIsabel.PintoCatarino@hcuge.ch

Co-investigators:

Pr Patrick Petignat, Gynecology service and Surgical Gynecologic Oncology Unit, HUG

Patrick.Petignat@hcuge.ch

PD, Dr Pierre Vassilakos, Geneva Foundation for Medical Education and Research, Geneva.

pierrevassilakos@bluewin.ch

Aline Bilancioni, referent nurse in colposcopy

Aline.Kindynis@hcuge.ch

Abstract

Background: The Xpert HPV test offers the opportunity of a point-of-care assay to detect high-risk human papillomavirus (hrHPV) infection. It is a non-batch, qualitative real-time Polymerase Chain Reaction (PCR) assay. The Xpert HPV is performed with specimen transported in a methanol-water medium (PreservCyt). However, liquid storage is not practical.

Objective: Evaluate the feasibility of vaginal self-sampling using dry swabs for hrHPV-testing with the Xpert HPV

Material and method: Two vaginal specimens will be collected for HPV testing and stored in different mediums for each woman. Women will firstly be asked to perform a Self-HPV using a dry swab (S-DRY) and then the physician or nurse will perform a cervical collection immersed in PreservCyt (dr-WET). HPV types will be identified by the Xpert HPV assay. The remaining sample immersed in PreservCyt will be tested for HPV DNA using cobas HPV test. A sample of 124 patients will be included. Women will complete a self-administered questionnaire on demographics. Agreement between the two methods and HPV type will be measured using the kappa statistic (κ). Sensitivity and specificity will be calculated for each method, using the cobas HPV test results as reference.

Expected results: Dry vaginal swabs are a feasible and equivalent method to collect and store vaginal specimens for testing with the Xpert HPV.

Key words: cervical cancer, dry swabs, human papillomavirus (HPV), self-sampling, Xpert HPV assay

1. Background

Recent development of tests for high-risk human papillomavirus (hrHPV) infection has created an important change in our understanding of cervical cancer screening.

Overwhelming evidence from several randomized trials have shown that HPV screening is more effective than cytology in preventing cervical cancer. It demonstrates better sensitivity and allows less frequent screening[1, 2].

Besides developed countries, which progressively are incorporating HPV testing in their national cervical cancer-screening program and updating their current guidelines[3], developing countries, following the recommendations of the World Health Organization, are evaluating the HPV testing as a primary screening tool[4].

HPV testing is associated with decreased cervical cancer-related mortality[5, 6] and it gives the possibility to use a self-vaginal sampling (self-HPV), which is considered as accurate as physician-cervical sampled HPV tests[7].

Not only for low resource settings, but equally for developed countries like Switzerland, the introduction a point-of-care, rapid, non-batch assay facilitating same-day screen and management strategies is essential. This approach would minimize repeat visits, allowing therefore, most of the eligible women to participate in the program. The ideal system should be modular and easily integrated into modest-income settings.

Furthermore PCR-based hrHPV tests with a higher analytic sensitivity should be preferred because they ensure similar accuracy between clinician-collected and self-collected samples[7].

Such a point-of-care testing seems to be the Xpert HPV assay (Cepheid, Sunnyvale, CA, USA), a non-batch and qualitative real-time PCR assay for the detection of hrHPV DNA. The assay is formatted in a single-use test cartridge. A single test can be completed in 1 hour, allowing same-day screen-and-treat, which reduces the potential loss to follow-up. The developed system provides a modular flexibility ranging from 1 to 80 test processing modules able to deliver from one to 471 HPV test a day.

Until now the Xpert HPV test was evaluated with specimens previously collected into ThinPrep (Hologic) vials containing a methanol-water solution (PreservCyt transport medium)[8]. This approach may be impractical and unavailable, because of flammability, toxicity and cost, especially in low resource settings. In developed countries, women have expressed concerns for liquid transport medium, thinking that the test quality is reduced by accidentally spilling out some of the transport medium[9, 10].

In a previous self-HPV study we found that swabs transported in a dry state were as accurate as those obtained with swabs shipped in a wet transport medium, in terms of quality of results[11].

The possibility of using self-obtained specimens stored at ambient temperature without transport media would clearly enhance and simplify the utility of Self-HPV. Moreover it reduces the costs of the method, which might be attractive for a point-of-care strategy.

The objective of our study is to compare performance diagnosis for hr-HPV infection of Self-HPV using dry swabs (S-DRY) and Physician collected sample with swab immediately immersed in PreservCyt (dr-WET). The comparison will be carried out after testing with the Xpert HPV.

If the S-DRY approach with the Xpert HPV assay is feasible, it will assist the implementation of a cost-effective screening strategy worldwide; in developing countries, by overcoming material and human barriers and minimizing need for repeat visits, preventing loss of follow-up; and in developed countries by potentially increasing the participation rate in already well established screening programs by giving women alternatives for screening, which might be more adapted to their busy schedule or budget, encouraging them to get screened.

2. Objective

Evaluate analytic performance of two transportation and storage devices: Physician collected sample with swab immediately immersed in PreservCyt (dr-WET) versus Self-vaginal sampling with dry swab (S-DRY).

3. Material and method

Inclusion Criteria:

- ≥ 18 years
- Attending colposcopy clinic
- Understands study procedures and accepts voluntarily to participate by signing the informed consent form (ICF)

Exclusion Criteria:

- Pregnancy
- Previous Hysterectomy

Study design:

Women will be invited to perform two samplings for HPV testing. Women will firstly be asked to perform a Self-HPV using a dry swab (S-DRY) and then the physician or nurse will perform a cervical collection immersed in PreservCyt (dr-WET). All specimens will be tested for the same pathogens (HR-HPV) using the same diagnostic test (Xpert HPV). Later, the remaining sample immersed in PreservCyt will be tested for HPV DNA using cobas HPV test.

Study procedure:

A research nurse will give instructions to the patients and ICF will be obtained. For specimen collection, participants will be instructed to wash their hands before the procedure. Each participant will receive a package containing specimen collection kit. Recommendations will be to hold the swab by the end of the handle, to insert the swab into the vagina, avoiding contact with the external genitalia, until they meet resistance (at least 6 cm). Once they meet resistance, they shall gently turn the swab three to five times. Subsequently the swab shall be inserted in inside a plastic sleeve (S-DRY). Then, the physician or nurse will perform a cervical collection immersed in PreservCyt (dr-WET). In case that the patient is intended to perform a routine cytology during consultation, this cervical collection for the study, will always be performed in second. During colposcopy consultation, the physician or nurse will proceed with the routine consultation. We will look for the histology and cytology results from patients who have done recently a biopsy.

At the end, women will complete a self-administered questionnaire on demographics.

HPV analysis:

Dry swabs (S-DRY samples) will be placed and rinsed into tubes with 3 ml of sterile phosphate-buffered saline (PBS) or NaCl 0.9%, and the tubes will be vortexed for 3×15 sec. Then, 1 ml of each sample will be transferred to the cartridge and will be run on a four-module GeneXpert machine. Tubes containing dr-WET samples (3 ml PreservCyt medium) will be also vortexed for 3×15 sec. Then, 1 ml of each sample will be transferred to the cartridge and will be run on the GeneXpert machine.

S-Dry and dr-WET aliquots will be run simultaneously to the GeneXpert machine. The remaining sample immersed in PreservCyt (2ml) will be tested for HPV DNA using cobas HPV test. Once a valid result is obtained, the dry swab specimen will be discarded.

Xpert HPV analysis: The Xpert HPV analysis performed consists of real time PCR, using as internal assay control for specimen adequacy, the detection of a human reference gene (HMBS [hydroxymethylbilane synthase]) and an internal Probe Check Control (PCC). The PCC verifies reagent rehydration, PCR tube filling in the cartridge, probe integrity, and dye stability.

This test includes reagents for the simultaneous detection of 14 hrHPV types (HPV16, 18, 31, 33, 35, 39, 45, 51, 52, 56, 58, 59, 66 and 68).

The assay utilizes multiple fluorescent channels for the detection of individual types of HPV, groups of HPV, and the human reference gene. Each fluorescent channel has its own cutoff parameters for target detection/validity. If sufficient signal is detected by the human reference gene, the assay results are reported as an overall “positive” if any type of targeted HPV is

detected, but, additionally HPV16 and pooled HPV18/45 and, collectively, the other high-risk HPV types detected by the assay are reported specifically as “positive” or “negative.”

Cobas test: samples will be analyzed by qualitative PCR using the cobasR4800 System according to the algorithm of Roche Diagnostics. This test allows the specific identification HPV 16 and HPV 18 and detects all other high-risk types (31, 33, 35, 39, 45, 51, 52, 56, 58, 59, 66 and 68) at clinically relevant infection levels.

Statistical analysis:

The tests will be based on the binomial distribution and the exact confidence intervals (CI) will be given. Kappa values will be calculated using standard methods. The agreement will be 0 when the amount of agreement is what would be expected by chance and 1 when there is perfect agreement. All comparisons will be two-sided, and P values less than 0.05 will be considered statistically significant.

The Xpert HPV uses as internal assay control for specimen adequacy the detection of a human reference gene (HMBS) where threshold (Ct) values reflect the relative amount of cells in the sample. A lower Ct-value represents a relatively higher number of cells. Thus, the mean Ct-values for the HMBS adequacy-test will be calculated for the two sampling modalities.

Agreement between the S-DRY and dr-WET samples concerning HPV types and HPV positivity will be measured using the kappa statistic (κ).

Sensitivity and specificity will be calculated for each method, using the cobas HPV test results as reference. Cytological results will also be used as a reference.

We will also analyze invalid test results using the Xpert HPV test and the delay between self collection and HPV analysis.

A sample size of 150 women will be sufficient to provide a 10% precision to estimate the kappa coefficient, if the κ is 50% (worst case scenario, as the precision will be better if the κ is lower or higher than 50%). Assuming a 40% prevalence of the HPV infection in our selected population, the precision of other measures will be more or less 15%.

4. Samples storage

In case of eventual delays that do not allow HPV testing to be immediately ran, samples will be stored in the fridge (2°C) and they will be analyzed the day after at the latest. Delays between self collection and HPV analysis will be recorded for each patient.

All samples will be destroyed at the end of the study.

5. Ethical issues

The colposcopy consultation will not be altered by the study. Results of specimen's analysis will be discussed with the female patients.

6. Financial consideration

This study includes no charge for the patients and HUG. Financial support was obtained for a 20% research nurse for 9 months period (FRS 15'000) and to buy the Xpert HPV test (FRS 17'000)

7. Expected contribution

We use a cost-effective strategy that if it proves to be equivalent to the standard Xpert HPV test with PreservCyt, will contribute to the development and validation of this method for HPV screening, simplifying the actual procedure.

8. References

1 Arbyn Marc, Vaccine 2012 vol 30 F88-F89

2 Ronco G, Dillner J, Elfström KM, Tunesi S, Snijders PJ, Arbyn M, et al. Efficacy of HPV-based screening for prevention of invasive cervical cancer: follow-up of four European randomised controlled trials. The Lancet. 2013

3 Castle PE, de Sanjosé S, Qiao YL, Belinson JL, Lazcano-Ponce E, Kinney W. 2012. Introduction of human papillomavirus DNA screening in the world: 15 years of experience. Vaccine 30(Suppl 5):F117–F122. http://dx .doi.org/10.1016/j.vaccine.2012.05.071.

4 http://apps.who.int/iris/bitstream/10665/94830/1/978924 1548694_eng.pdf

5 Sherris, J., et al., *Evidence-based, alternative cervical cancer screening approaches in low-resource settings.* International perspectives on sexual and reproductive health, 2009. 35(3): p. 147-152.

6 Sankaranarayanan, R., et al., *HPV screening for cervical cancer in rural India.* New England Journal of Medicine, 2009. 360(14): p. 1385-1394.

7 Arbyn M, Verdoodt F, Snijders PJF, et al. Accuracy of human papillomavirus testing on self-collected versus clinician-collectedsamples: a meta-analysis. *Lancet Oncol* 2014; published online Jan 14. http://dx.doi.org/10.1016/S1470-2045(13)70570-9

8 Einstein MH, Smith KM, Davis TE, Schmeler KM, Ferris DG, Savage AH, Gray JE, Stoler MH, Wright TC Jr, Ferenczy A, Castle PE. *J Clin Microbiol.* 2014 Jun; 52(6):2089-95.Clinical Evaluation of the Cartridge-Based GeneXpert Human Papillomavirus Assay in Women Referred for Colposcopy.

9 Krech T, Castriciano S, Jang D, Smieja M, Enders G, Chernesky M: Detection of high risk HPV and Chlamydia trachomatis in vaginal and cervical samples collected with flocked nylon and wrapped rayon dual swabs transported in dry tubes. J Virol Methods 2009, 162:291–293.

10 Gustavsson I, Lindell M, Wilander E, Strand A, Gyllensten U: Use of FTA card for dry collection, transportation and storage of cervical cell specimen to detect high-risk HPV. J Clin Virol 2009, 46:112–116.

11 Isabelle Eperon, Pierre Vassilakos, Isabelle Navarria, Pierre-Alain Menoud, Aude Gauthier, Jean-Claude Pache, Michel Boulvain, Sarah Untiet and Patrick Petignat Randomized comparison of vaginal self-sampling by standard vs. dry swabs for Human papillomavirus testing. BMC Cancer 2013, vol. 13, p. 353

Annexes

Flowchart I

Female Patient

- **Attending Colposcopy clinic**
- **Complete eligibility criteria**
- **Completing an ICF**

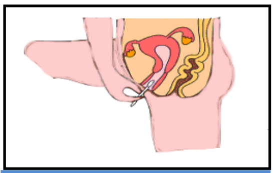

Female Patient
Self-DRY*

Gynecologist/ Nurse

Cervical sampling for HPV analysis**
Colposcopy (+- cytology¥ +- biopsy and CEC)

Specimens collected by the 2 methods will be tested for HR-HPV, using the same diagnostic test **(Xpert HPV)**

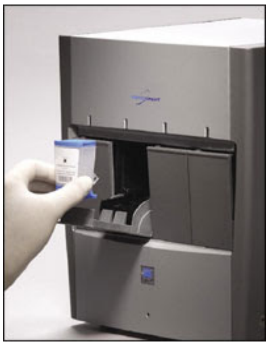

Residual PreservCyt sample (2ml) will be tested for HPV DNA using cobas HPV test.

* a) Unsupervised Self-vaginal sample with dry swab; b) delayed elution in PBS or Saline (NaCl 0.9%), (1ml).

** Physician's cervical sampling with swab immediately immersed in PreservCyt (1 ml);

¥ If the patient is intended to have a cytological examination during her normal consultation, the collection performed by the physician for HPV will always be done in second.
